# Anti-Leukemic Activity of Brassica-Derived Bioactive Compounds in HL-60 Myeloid Leukemia Cells

**DOI:** 10.3390/ijms232113400

**Published:** 2022-11-02

**Authors:** María Ángeles Núñez-Sánchez, María Antonia Martínez-Sánchez, Marina Verdejo-Sánchez, Paula García-Ibáñez, Alba Oliva Bolarín, Bruno Ramos-Molina, Diego A. Moreno, Antonio J. Ruiz-Alcaraz

**Affiliations:** 1 Obesity and Metabolism Laboratory, Biomedical Research Institute of Murcia (IMIB), 30120 Murcia, Spain; 2 Department of Biochemistry, Molecular Biology B and Immunology, School of Medicine, University of Murcia, Regional Campus of International Excellence “Campus Mare Nostrum”, 30100 Murcia, Spain; 3 Aquaporins Research Group, Department of Plant Nutrition, Centro de Edafología y Biología Aplicada del Segura (CEBAS), CSIC, Campus de Espinardo-25, 30100 Murcia, Spain; 4 Phytochemistry and Healthy Food Lab (LabFAS), Department of Food Science Technology, Centro de Edafología y Biología Aplicada del Segura (CEBAS), CSIC, Campus de Espinardo-25, 30100 Murcia, Spain

**Keywords:** *Brassica*, phytochemicals, leukemia, cancer, sulforaphane, phenolic compounds

## Abstract

Acute myeloid leukemia (AML) is a cancer of the myeloid blood cells mainly treated with chemotherapy for cancer remission, but this non-selective treatment also induces numerous side effects. Investigations with bioactive compounds from plant-derived foods against cancer have increased in the last years because there is an urgent need to search for new anti-leukemic agents possessing higher efficacy and selectivity for AML cells and fewer negative side effects. In this study, we analyzed the anti-leukemic activity of several phytochemicals that are representative of the major classes of compounds present in cruciferous foods (glucosinolates, isothiocyanates, hydroxycinnamic acids, flavonols, and anthocyanins) in the human acute myeloid leukemia cell line HL-60. Our results revealed that among the different Brassica-derived compounds assayed, sulforaphane (SFN) (an aliphatic isothiocyanate) showed the most potent anti-leukemic activity with an IC_50_ value of 6 µM in dose-response MTT assays after 48 h of treatment. On the other hand, chlorogenic acid (a hydroxycinnamic acid) and cyanidin-3-glucoside (an anthocyanin) also displayed anti-leukemic potential, with IC_50_ values of 7 µM and 17 µM after 48 h of incubation, respectively. Importantly, these compounds did not show significant cell toxicity in macrophages-like differentiated cells at 10 and 25 µM, indicating that their cytotoxic effects were specific to AML cancer cells. Finally, we found that these three compounds were able to induce the NRF2/KEAP1 signaling pathway in a dose-dependent manner, highlighting SFN as the most potent NRF2 activator. Overall, the present evidence shed light on the potential for using foods and ingredients rich in anticancer bioactive phytochemicals from *Brassica* spp.

## 1. Introduction

Myeloid leukemia is a type of cancer characterized by the proliferation of malignant myeloid-derived cells in the bone marrow. Acute myeloid leukemia (AML), like other cancers, presents a heterogeneous etiology that leads to different pathophysiology patterns, although its main features include a fast progression of the disease and a poor prognosis [1]. Currently, AML chemotherapy incorporates cytarabine, anthracycline, and cladribine individually or in combinations [1,2], as effective agents for cancer remission, but with numerous side effects because of their low selectivity for cancer cells [3,4,5]. Furthermore, in some cases, patients either do not respond to the used treatments or the effectiveness of these drugs is only partial and, thereby, a relapse of AML appears because of the presence of residual leukemic-resistant cells [6]. Thus, there is an urgent need for new anti-leukemic agents with better efficacy and selectivity for cancer cells and fewer negative side effects [7,8,9]. In this regard, dietary sources of phytochemicals and their bioactive metabolites are good candidates for searching for active agents in the treatment of leukemia [10,11,12,13,14]. 

In addition to essential nutrients, vegetables are rich sources of phytochemicals with potential health benefits. Specifically, among the most important vegetables are those belonging to the *Brassicaceae* family. This includes several crops, such as broccoli, cabbages, and radishes, with a high socioeconomic relevance [15]. They are known to be rich in phenolic compounds and other biomolecules such as glucosinolates (GSL). The latter are stable secondary metabolites that, by the action of the myrosinase enzyme (EC 3.2.1.147), are hydrolyzed producing a variety of bioactive compounds such as isothiocyanates (ITC) [16,17,18]. Remarkably, certain ITCs are able to induce Phase II detoxification enzymes and thus, exert anti-carcinogenic effects in a myriad of experimental models [19,20,21,22]. In addition, glycosylated and acylated derivatives of cinnamic acids, flavonols, and anthocyanins (mainly cyanidins) are the major and ubiquitous representative phytochemicals in cruciferous vegetables [23,24,25]. Emerging evidence has proposed that certain dietary bioactive compounds may have potential interest in cancer therapy. However, the antileukemic effects of bioactive compounds present in *Brassica* spp. remain poorly understood [26].

Accumulating evidence indicates that *Brassica* spp. phytochemicals may exert anti-tumoral activities by activating nuclear factor erythroid 2-related factor 2 (NRF2) which is a master modulator of redox homeostasis [27]. For example, sulforaphane (SFN), the bioactive ITC derived from the aliphatic glucoraphanin (GRA) and very abundant in cruciferous vegetables, has been shown to be a potent NRF2 activator with anti-carcinogenic properties in a number of experimental models [16,28,29]. On the other hand, the indole-3-carbinol (I3C), and phenolic acids (e.g., chlorogenic acid, CQA), or flavonoids (e.g., rutin), have been also tested in vitro and in vivo to explore their therapeutic potential in the treatment of leukemia, providing the basis for new alternative therapies [11,30,31,32]. However, the activity of *Brassica* spp. bioactive compounds other than SFN as NRF2 inducers in cancer cells remain largely unexplored.

In this work, we have analyzed the anti-leukemic activity of a set of phytochemicals representing the major classes of bioactive compounds in cruciferous foods (GSL, ITC, hydroxycinnamic acids, flavonols, and anthocyanins) as well as the activity of the most potent anticancer compounds as NRF2 inducers in human AML cells.

## 2. Results

### 2.1. Analysis of the Anti-Leukemic Potential of Brassica spp. Phytochemicals in HL-60 Cells

**Figure 1:**
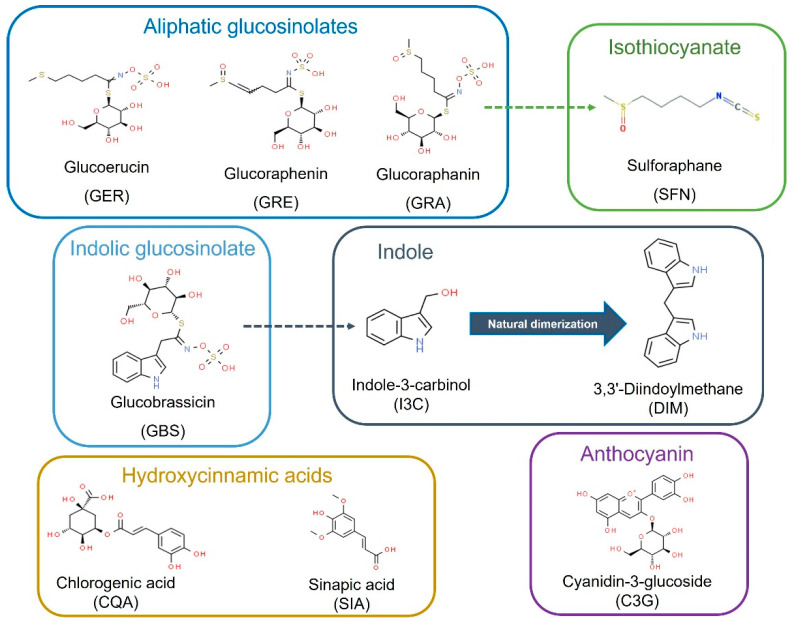
Bioactive compounds selected for this study as commonly present and characteristic of *Brassica* spp. foods and ingredients. Dash arrows connect precursors and bioactive metabolites.

The chemical and structural properties of the natural bioactive compounds included in this study are shown in Figure 1. The screening of the potential anti-leukemic activity of the compounds was initially measured by MTT assay at relatively high doses (10 μM, 25 μM, and 50 µM) of all the compounds for three time-points: 24, 48, and 72 h. 

**Figure 2:**
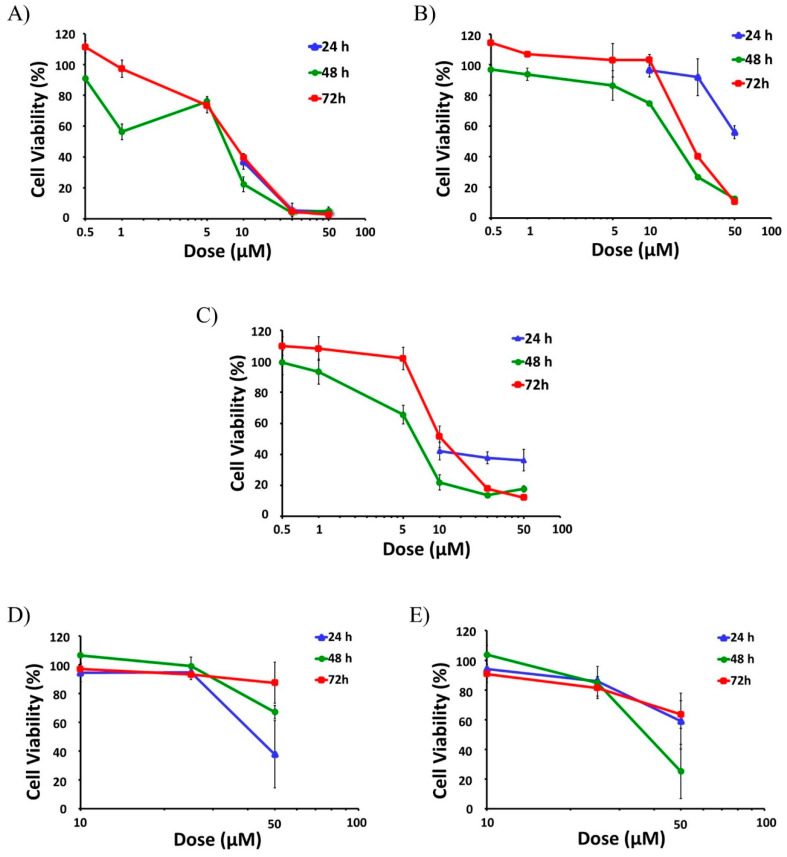
**Anti-leukemic effect of *Brassica* spp. phytochemicals in HL-60 cells.** The screening of the potential anti-leukemic activity of the *Brassica* spp. phytochemicals were initially done with high doses (10 μM, 25 μM, and 50 µM) of all compounds for 24, 48, and 72 h. Then, the selected more active compounds (SFN, CQA, and C3G) were subjected to further dose-response analysis by testing lower doses (0.5 μM, 1 μM and 5 μM) at 48 and 72 h. Data represents % of cell viability ± SD of HL-60 human leukemia cells in the presence of active Brassica-derived compounds measured by MTT assays (*n* = 3, three independent experiments performed in triplicate). (**A**) Anti-leukemic potential of SFN. (**B**) Anti-leukemic potential of CQA. (**C**) Anti-leukemic potential of C3G. (**D**) Anti-leukemic potential of I3C. (**E**) Anti-leukemic potential of DIM. SFN, sulforaphane; CQA, Chlorogenic acid; C3G, cyanidin-3-glucoside chloride; I3C, indole-3-carbinol; DIM, 3,3′-Diindolmethane.

Then, selected active compounds were subjected to further dose-response analysis by adding lower doses (0.5 μM, 1 μM, 5 μM) to the assays at 48 and 72 h (Figure 2). Among all the tested compounds SFN had the highest effect on cell viability, as it was able to decrease the viability of the active proliferative cells at the two higher doses tested (25 µM and 50 µM), reaching cytotoxicity levels above 95% for all time points (24, 48 and 72 h) (Figure 2A). In fact, SFN already reduced the viability of the cells to levels below 50% for all time points with a 10 µM dose (Figure 2A). 

Dose-response assays performed at 48 and 72 h showed that IC_50_ values of SFN were 5.9 µM and 11.3 µM, respectively (Table 1). On the contrary, other aliphatic GSLs such as GRA, GRE, and GER did not show any significant effects on HL-60 viability, even at 50 µM [33]. 

CQA also showed a strong anti-AML effect, leading to a significant reduction of cell viability at all tested times (Figure 2B). This effect was especially remarkable after 48 and 72 h treatments, with the viability reduced to 13% and 11% respectively for the higher dose and even lower than 50% with the 25 µM treatment (Figure 2B), and IC_50_ values of 17.4 and 28.9 µM (Table 1). By contrast, sinapic acid (SIA) did not have any effect whatsoever on cell viability in HL-60 cells [33].

The treatment of HL-60 cells with cyanidin-3-glucoside chloride (C3G), an anthocyanin, presented a mild but constant effect already at 24 h, showing cell viability levels around 40% when treated with 10–50 µM doses (Figure 2C). This effect was even more potent at longer times (48 h and 72 h) for the higher doses (25 and 50 µM), lowering cell viability below 20% (Figure 2C). IC_50_ values of C3G were 7.0 µM for the 48-h treatment and 21.9 µM for 72 h (Table 1).

In the case of the indolic group of compounds, including glucobrassicin (GBS), I3C, and DIM, only I3C and DIM showed significant antiproliferative effects. While I3C was able to reduce the viability of the cells down to 38%, but just at the shorter time (24 h) with the maximum dose (Figure 2D), DIM showed a mild effect with the higher dose at all the three time-points assayed, reaching a maximum cytotoxicity of 75% at 48 h (25% viability), (Figure 2E). GBS did not show any effect on cell viability in any experimental condition.

### 2.2. Effects of Brassica spp. Phytochemicals in the Cell Viability of Macrophage-like Differentiated HL-60 Cells

The previously described viability results were further compared to those obtained with differentiated cells to verify the specificity of the anti-tumor/cytotoxic effect of the active compounds upon the proliferative leukemia cells [34,35]. The treatment of differentiated HL-60 cells with the active compounds did not show significant differences in cell viability with any of the doses at 48 h, with the only exception of SFN at concentrations higher than 10 µM, for which it produced a significant cytotoxic effect (Table 2). 

Nonetheless, the administration of SFN at 10 µM to differentiated cells did not cause significant cytotoxicity. Overall, these results indicate that the cytotoxic effect of the compounds was remarkably higher in proliferative leukemia cells with respect to differentiated macrophage-like cells.

### 2.3. Potential of Brassica spp. Phytochemicals as NRF2 Inducers in Human Acute Myeloid Leukemia HL-60 Cells

**Figure 3:**
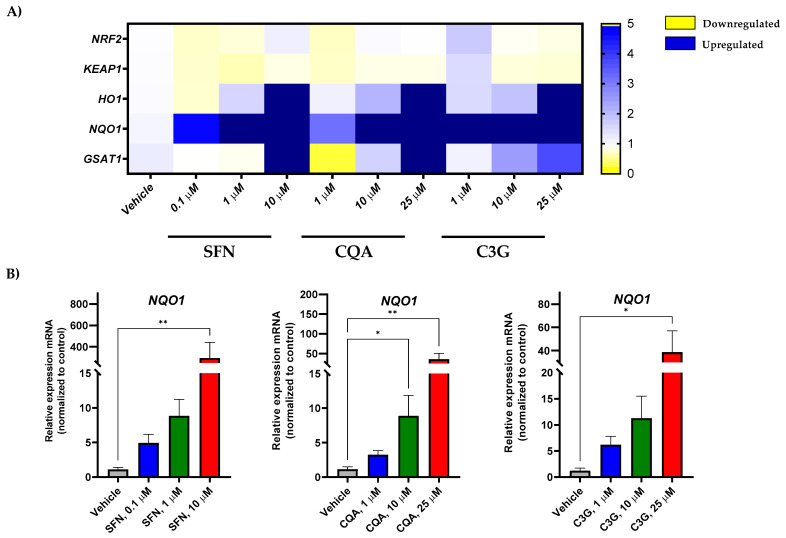
***Brassica* spp. phytochemicals modulate gene expression levels of Nrf2-target genes in the undifferentiated HL-60 cell line.** (**A**) Human acute myeloid leukemia cell line HL-60 gene expression profile in response to SFN, CQA, and C3G exposure at different concentrations by qRT-PCR. Represented as the fold change between condition and the vehicle group. Values outside the range represented in dark blue; (**B**) Significant changes in oxidative stress genes by RT-qPCR. All values are represented as the fold change of relative RNA expression between the condition and the vehicle group. Statistical analyses were performed using Kruskal- Kruskal–Wallis one-way analysis of variance and Dunn’s *post hoc* analysis. All values are given as mean ± SD. * *p* < 0.05, ** *p* < 0.01. SFN, sulforaphane; CQA, Chlorogenic acid; C3G, cyanidin-3-glucoside chloride; I3C, indole-3-carbinol; DIM, 3,3′-Diindolmethane.

In order to test the effect of the most potent anti-leukemic compounds (SFN, CQA, and C3G) on NRF2 activation in human acute myeloid leukemia HL-60 cells, we performed gene expression analysis by RT-qPCR of NFR2 (encoded by the *NFE2L2* gene) and related genes. As shown in Figure 3A and Figure S1, SFN, CQA, and C3G were able to markedly induce the expression levels of the NRF2-target genes including *HMOX*, *NQO1,* and *GSTA1* in a dose-dependent fashion. 

Furthermore, none of these bioactive compounds was able to increase the gene expression of other antioxidant enzymes such as *CAT*, *SOD1,* or *GPX1* (Figures S2 and S4), suggesting that these Brassica-derived phytochemicals are able to specifically induce the NRF2 pathway, but not other enzymes involved in redox homeostasis [33,36]. Remarkably, none of the tested compounds overactivated NRF2-dependent genes in HL-60 macrophage-like differentiated cells (Figure 3 and Figure S3), suggesting that *NFE2L2* induction was specific to cancer cells. 

## 3. Discussion

Previous epidemiological studies have shown an inverse association between the consumption of vegetables from the *Brassicaceae* family and the risk of developing lung [37], breast [38], and prostate cancer [39]. In this context, phytochemicals (GSL and phenolic compounds) from cruciferous foods, have been largely studied for their anticancer potential [40,41,42]. However, knowledge about the anti-tumoral activity of cruciferous-derived bioactive compounds against AML is still scarce. Thus, in this study, we performed an in vitro study focusing on the potential anti-tumor/cytotoxic activity of a series of *Brassica* spp. natural compounds in the human AML cell line HL-60. Our results revealed that among 10 different compounds tested, the treatment of HL-60 cells with SFN, CQA, and C3G, displayed remarkable anti-AML activity, as they were able to significantly reduce the viability of undifferentiated leukemia cells with low IC_50_ values.

Several in vitro and in vivo studies have allowed for the identification of several mechanisms involved in the anti-carcinogenic effect of SFN including cell cycle arrest, induction of apoptosis, inhibition of angiogenesis, and induction of Phase II and antioxidant enzymes [14,43]. In this study, we found that, among the different compounds tested, SFN was the one displaying the most potent cytotoxic effect, which is in line with previous results from other authors [44,45]. Similarly, the anti-tumoral activity of anthocyanins and hydroxycinnamic acids was evaluated against AML exerting a synergic activity with antineoplastic drugs [11]. Our study revealed that the anthocyanin C3G and the hydroxycinnamic acid CQA were able to exert potent cytotoxic effects at relatively low concentrations after 48 h. These results are partially in agreement with those from other authors. For instance, a previous study of the anti-carcinogenic effect of C3G cells showed that this compound inhibited the proliferative capacity of HL-60 cells through p53-independent apoptosis induction together with an increased cytodifferentiation [46]. However, the concentrations needed to reach such capacity were considerably higher (>100 µM) than the ones tested in this work [46]. Likewise, there is certain controversy about the effect of the CQA acid on HL-60 cell viability. Thus, similar to some previous results [47], but in contrast to others [48], our data showed that CQA also has a mild, but significant, cytotoxic effect on human HL-60 leukemia cells. Finally, from the other 7 compounds tested, only the indolic metabolites I3C and 3,3′-Diindolmethane (DIM) showed a mild but significant cytotoxic effect after 24 h. 

The anti-carcinogenic activity of ITC has been largely studied over the last 30 years [27]. Importantly, a number of studies have proven that many of these compounds could exert these effects through the activation of the NRF2 pathway [49]. NRF2, which is encoded by the *NFE2L2* gene, is a master regulator of the antioxidant response that involves not only antioxidant enzymes but also phase II detoxifying enzymes [50]. Hence, here we evaluated the impact of the selected compounds on genes involved in the NRF2 pathway such as the *NFE2L2* modulator *KEAP1* and the downstream products *HMOX1*, *NQO1*, and *GSTA1*. Our results showed that the expression of NRF2-regulated genes increased in a dose-dependent manner. However, although a tendency was observed in *HMOX1* and *GSTA1*, this was not significant enough. On the other hand, the expression of the phase I oxidation enzyme gene *NQO1* was significantly induced by all three compounds at the highest concentration (Figure 3). Although activation of *NQO1* can act as a tumor suppressor, a “Janus” effect of NQO1 in cancer has been suggested [51]. Thus, while some studies have described an anti-tumoral effect through quinone detoxification, superoxide scavenging, and antioxidant maintenance [52], other authors have found that cancer cells such as melanoma, colorectal cancer, or breast cancer overexpress this enzyme, which confers malignant cells protection against oxidative stress [53,54,55]. Since the anti-leukemic effects occurred in parallel to a significant induction of *NQO1* mRNA levels, NQO1 could have anti-cancer activity in this human AML model.

## 4. Materials and Methods

### 4.1. Compounds

The GSL glucoraphanin (GRA), glucoerucin (GER), and glucobrassicin (GBS) were all obtained of high-purity standards from Phytoplan Diehm and Neuberger GmbH (Heidelberg, Germany). SFN, I3C, and DIM, were all obtained from LKT Laboratories, Inc. (St. Paul, MN, USA). The hydroxycinnamic acids CQA and SIA), as well as the anthocyanin C3G, were obtained from Phytoplan Diehm and Neuberger GmbH (Heidelberg, Germany).

### 4.2. Preparation of Compounds for In Vitro Assays

The stock solution of each compound was prepared in phosphate-buffered saline (PBS) (Biowest, Bradenton, FL, USA) at a concentration of 1 mg/mL except for SFN. Due to the low solubility of SFN in aqueous solutions, this was first dissolved in dimethyl sulfoxide (DMSO) at 10 mg/mL and then diluted in PBS at a 1:10 ratio. This solution represents 1 mg/mL of SFN with 10% DMSO and was used to prepare successive solutions at the appropriate concentrations in a complete culture medium, prior to being used in the in vitro assays. The media of cells exposed to the highest concentration of SFN contained 0.09% *v/v* DMSO. Compound solutions were added to the cells to obtain the final assay concentrations.

### 4.3. Cell Culture

The human acute myeloid leukemia cell line HL-60 (ATCC^®^ CCL-240™) was used to test the *Brassica* spp. phytochemicals. Maintenance of the cells was performed as previously described [33]. Briefly, cells were grown in RPMI-1640 medium (Biowest) supplemented with 10% fetal bovine serum (FBS; Gibco, Thermo Fisher Scientific, Madrid, Spain) and 1% penicillin/streptomycin (Gibco, Thermo Fisher Scientific, Madrid, Spain), at 37 °C with 5% CO_2_ and 90% humidity. All in vitro assays were performed after passage number 5 and before passage number 20 with cells growing at an exponential ratio.

### 4.4. In Vitro Cytotoxicity Assays

The anti-tumor activity of the compounds was evaluated by the MTT (3-(4,5-dimethylthiazol-2-yl)-2,5-diphenyltetrazolium bromide) viability assays as described previously [32,33]. In brief, HL-60 cells were cultured at a concentration of 10^5^ cells/mL (exponential growth rate) in 96-well plates containing 200 μL of cell culture medium and exposed to different doses of the tested compounds (0.5 μM, 1 μM, 5 μM, 10 μM, 25 μM, and 50 μM) for 24, 48, and 72 h. Then, MTT (Thermo Fisher) was added at a final concentration of 483 μM (0.2 mg/mL) and cells were incubated for 1 h at 37 °C and 5% CO_2_. Afterward, cells were lysed using an acidified isopropanol solubilization solution containing 0.04 M hydrochloric acid and 0.1% NP-40 detergent to release the formazan. Finally, the absorbance was measured at 550 nm using a SPECTROstar Nano (BMG Labtech, Germany) plate reader. The compound cytotoxicity was normalized to the control conditions (untreated with an equivalent dose of vehicle (DMSO) when applicable). 

In order to test the specificity of the compounds as potential anti-tumor/cytotoxic agents with no collateral negative effects upon non-proliferative cells, the series of compounds were also assayed in an in vitro model of human macrophage-like cells derived from the same HL-60 cell line. To obtain these differentiated macrophage-like cells, HL-60 human leukemia cells growing at an exponential rate in suspension were exposed to a dose of 10 ng/mL of phorbol myristate acetate (PMA) (Sigma-Aldrich, Merck, Análisis Vínicos SL, Tomelloso, Spain) for 24 h in 96-well plates at a cell density of 0.2·10^6^ HL-60 cells/well at 37 °C and 5% CO_2_. Cells were then incubated in culture media without PMA for another 24 h in the same culture conditions. After differentiation and resting periods, differentiated cells were treated for 48 h with compound solutions at different concentrations. Finally, MTT assays were performed as previously explained to measure cell viability. All experiments were performed in triplicate and in three independent experiments.

### 4.5. Gene Expression Assays

Total RNA was extracted from HL-60 cells using the GenElute Mammalian Total RNA Miniprep kit (Sigma) following the manufacturer’s instructions. RNA was eluted in RNAse-free water and checked for concentration and purity using the Nanodrop spectrophotometer system (ND-100 3.3. Nanodrop Technologies, Thermo Scientific, Madrid, Spain). Only samples with a ratio of Abs260/Abs280 between 1.8 and 2.1 were used for gene expression assays. cDNA was generated using a high-capacity RNA-to-cDNA kit (Thermo Fisher Scientific, Madrid, Spain) according to the manufacturer’s protocol. PCR amplification was carried out with PowerUp SYBR Green PCR Master Mix (Applied Biosystems, Waltham, MA, USA) using a Fast 7500 Real-Time instrument (Applied Biosystems, Waltham, MA, USA). The sequences of the primers are presented in Table S1. The obtained data were analyzed using the 7500 SDS software (Applied Biosystems, Waltham, MA, USA). Actin beta (*ACTB*) and Glyceraldehyde 3-phosphate dehydrogenase (*GAPDH*) were used as reference genes. Both were considered suitable reference genes for gene expression normalization based on geNorm and/or Bestkeeper stability analysis [34,35,36]. The gene-stability measures were performed using the RefFinder tool [36]. Relative expression was calculated using the 2^−ΔΔCt^ method. All experiments were performed in triplicate and in three independent experiments.

### 4.6. Statistical Analysis

Data were analyzed using the Prism 9.0 program (GraphPad Software Inc., La Jolla, CA, USA) using the Kruskal–Wallis one-way analysis of variance on ranks test followed by Dunn’s *post hoc* analysis. All values are given as mean ± SD. A level of *p* < 0.05 was considered statistically significant.

## 5. Conclusions

In this study, the bioactive compounds induced significant cytotoxic effects on the AML human cell line HL-60. These bioactive compounds have been described as anti-inflammatory agents [56], and therefore, they could not only contribute to the stop the progression of carcinogenesis but also reduce systemic inflammation in patients with cancer. Despite these promising results, further research is guaranteed for the evaluation of ingredients and foods rich in bioactive compounds from *Brassica* spp. (glucosinolates/isothiocyanates and phenolic compounds) to validate these beneficial effects by the bioaccessible and bioavailable metabolites ingested, going from preclinical models to nutritional interventions in cancer and chronic pathologies.

## Figures and Tables

**Table 1:** IC_50_ values of active cytotoxic compounds.

IC_50_ (μM)		
**Compound**	Treatment Time	
	48 h	72 h
**SFN**	5.9 ± 0.3	11.3 ± 0.5
**CQA**	17.4 ± 0.7	28.9 ± 0.7
**C3G**	7.0 ± 1.0	21.9 ± 0.3

Results represent IC_50_ values (µM) as mean ± SD following 48 and 72 h exposure of proliferative HL-60 cells to different doses (0.5 μM, 1 μM, 5 μM, 10 μM, 25 μM, and 50 μM) of active phytochemicals obtained from the MTT assay (*n* = 3). IC_50_ values were not determined for those compounds that did not reduce cells viability below 50%. SFN, sulforaphane; CQA, Chlorogenic acid; C3G, cyanidin-3-glucoside chloride; I3C, indole-3-carbinol; DIM, 3,3′-Diindolmethane.

**Table 2:** Effect of active cytotoxic compounds upon the viability of HL-60 differentiated cells.

	Viability (%)		
**Compound**	Dose (µM)		
	10	25	50
**SFN**	98.2 ± 12.1	87.6 ± 6.8	70.0 ± 17.5 *
**CQA**	113.5 ± 19.2	114.4 ± 20.6	108.7 ± 11.8
**C3G**	113.7 ± 24.4	108.1 ± 7.4	107.2 ± 12.8
**I3C**	93.6 ± 8.0	99.5 ± 5.4	95.1 ± 10.4
**DIM**	101.5 ± 22.7	117.7 ± 27.2	104.8 ± 13.5

Values are expressed as the mean ± SD following a 48-h exposure of HL-60 cells differentiated cells to the active cytotoxic compounds obtained from three different MTT assays (*n* = 3). * *p* < 0.05. SFN, sulforaphane; CQA, Chlorogenic acid; C3G, cyanidin-3-glucoside chloride; I3C, indole-3-carbinol; DIM, 3,3′-Diindolmethane.

## Data Availability

Not applicable, data would be available from coauthors upon request.

## Supplementary Materials

The following supporting information can be downloaded at: https://www.mdpi.com/article/10.3390/ijms232113400/s1.
