# Supplementary material for: Anti-Leukemic Activity of Brassica-Derived Bioactive Compounds in HL-60 Myeloid Leukemia Cells
Source: Int J Mol Sci. 2022 Nov 2;23(21):13400. doi: 10.3390/ijms232113400 (PMC9657236; doi:10.3390/ijms232113400)
Supplement: Supplementary file 1 [file ijms-23-13400-s001.zip › ijms-1990416-supplementary.pdf]

Table S1. Primer sequences

Gene name	Official gene symbol[a]	Alias symbol	Gene ID[b]	Forward	Reverse
NFE2 like bZIP transcription factor 2	*NFE2L2*	*NFR2*	4780	CGTTTGTAGATGACAATGAGG	AGAAGTTTCAGGTGACTGAG
Kelch like ECH associated protein 1	*KEAP1*	*INrf2, KLHL19*	9817	GCACAACTGTATCTATGCTG	CTCCAAGGACGTAGATTCTC
Heme oxigenase 1	*HMOX1*	*HO-1*	3162	CAACAAAGTGCAAGATTCTG	TGCATTCACATGGCATAAAG
NAD(P)H quinone dehydrogenase 1	*NQO1*	*DHQU*	1728	AGTATCCACAATAGCTGACG	TTTGTGGGTCTGTAGAAATG
Glutathione S-transferase alpha 1	*GSTA1*	*GSTA-1, GST2, GTH1*	2938	AGGTATAGCAGATTTGGGTG	AAGACTTTTTCAAAGGCAGG
Catalase	*CAT*	-	847	AGAGAAATCCTCAGACACATC	CAGCTTGAAAGTATGTGATCC
Superoxide dismutase	*SOD1*	*ALS, SOD*	6647	GAGCAGAAGGAAAGTAATGG	GATTAAAGTGAGGACCTGC
Glutathione peroxidase 1	*GPX1*	*GPx1*	2876	CTACTTATCGAGAATGTGGC	CAGAATCTCTTCGTTCTTGG
Glyceraldehyde-3-phosphate dehydrogenase	*GAPDH*	*G3PD*	2597	TCCACCACCCTGTTGCTGTA	ACCACAGTCCATGCCATCAC
Actin beta	*ACTB*	-	60	GATCAAGATCATTGCTCCTC	TTGTCAAGAAAGGGTGTAAC

[a]: HGNC, Hugo Gene Nomenclature Committee.

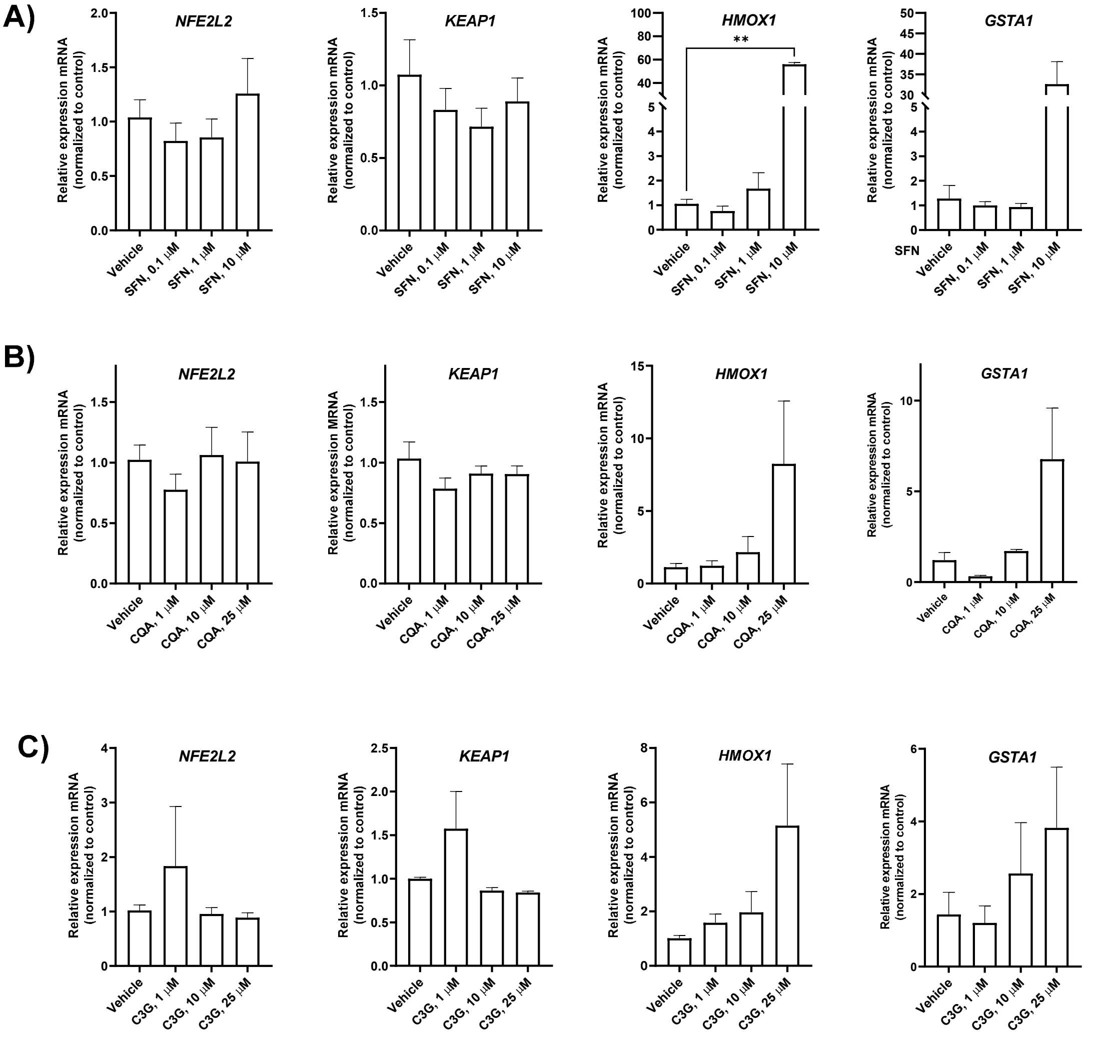

Figure S1. Changes in expression of NRF-2 pathway genes in undifferentiated HL-60 cell line exposed to different concentrations of A) Sulforaphane (SFN); B) Chlorogenic acid (CQA); and C) Cyaniding-3glc (C3G). All values are represented as the fold change of relative RNA expression between the condition and the vehicle group. Statistical analyses were performed using Kruskal- Kruskal–Wallis one-way analysis of variance and Dunn's *post hoc* analysis. All values are given as mean ± SD. ** $p < 0.01$

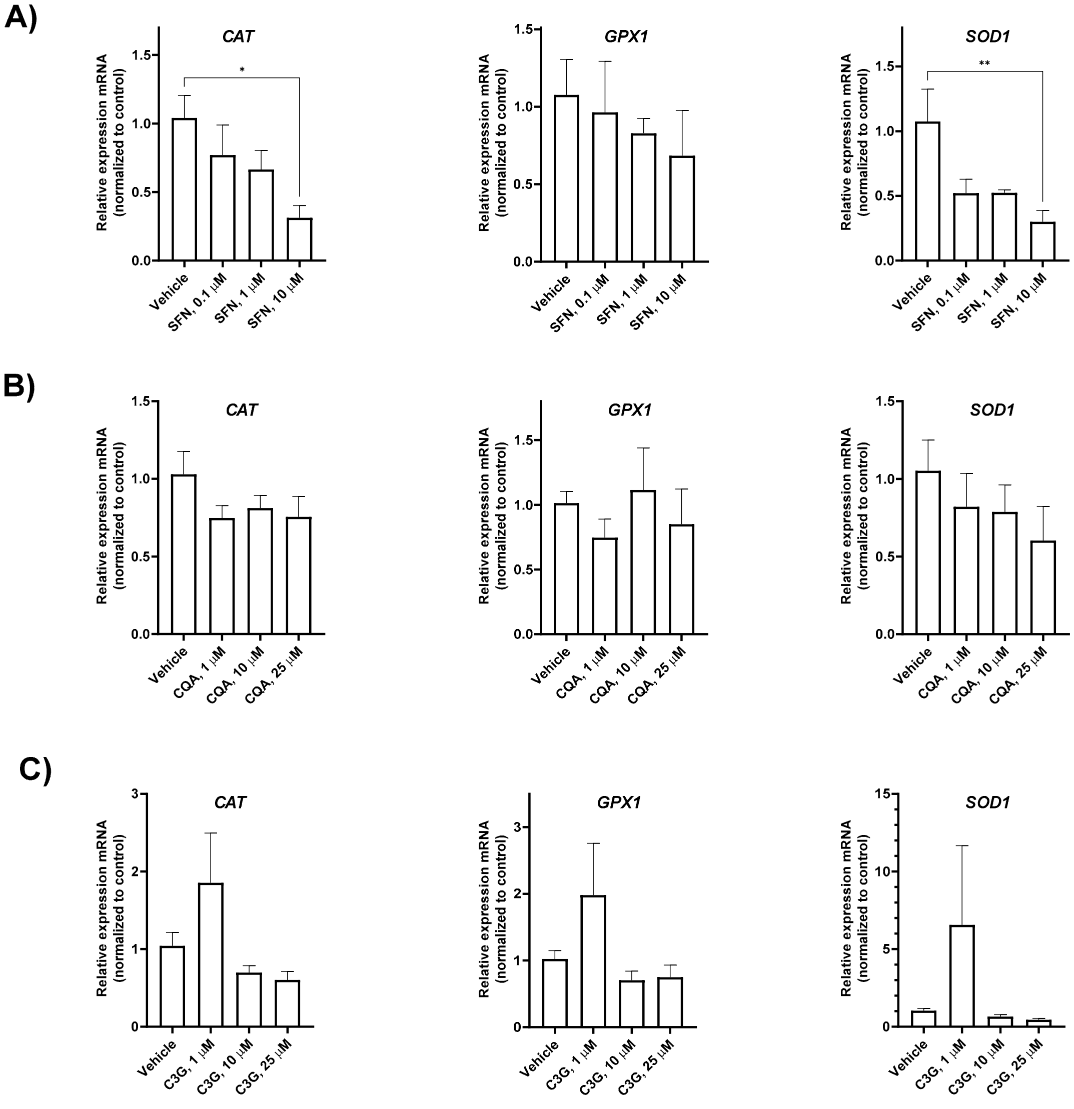

Figure S2. Changes in expression of antioxidant enzyme genes in undifferentiated HL-60 cell line exposed to different concentrations of A) Sulforaphane (SFN); B) Chlorogenic acid (CQA); and C) Cyaniding-3glc (C3G). All values are represented as the fold change of relative RNA expression between the condition and the vehicle group. Statistical analyses were performed using Kruskal- Kruskal–Wallis one-way analysis of variance and Dunn's *post hoc* analysis. All values are given as mean ± SD. ** $p < 0.01$

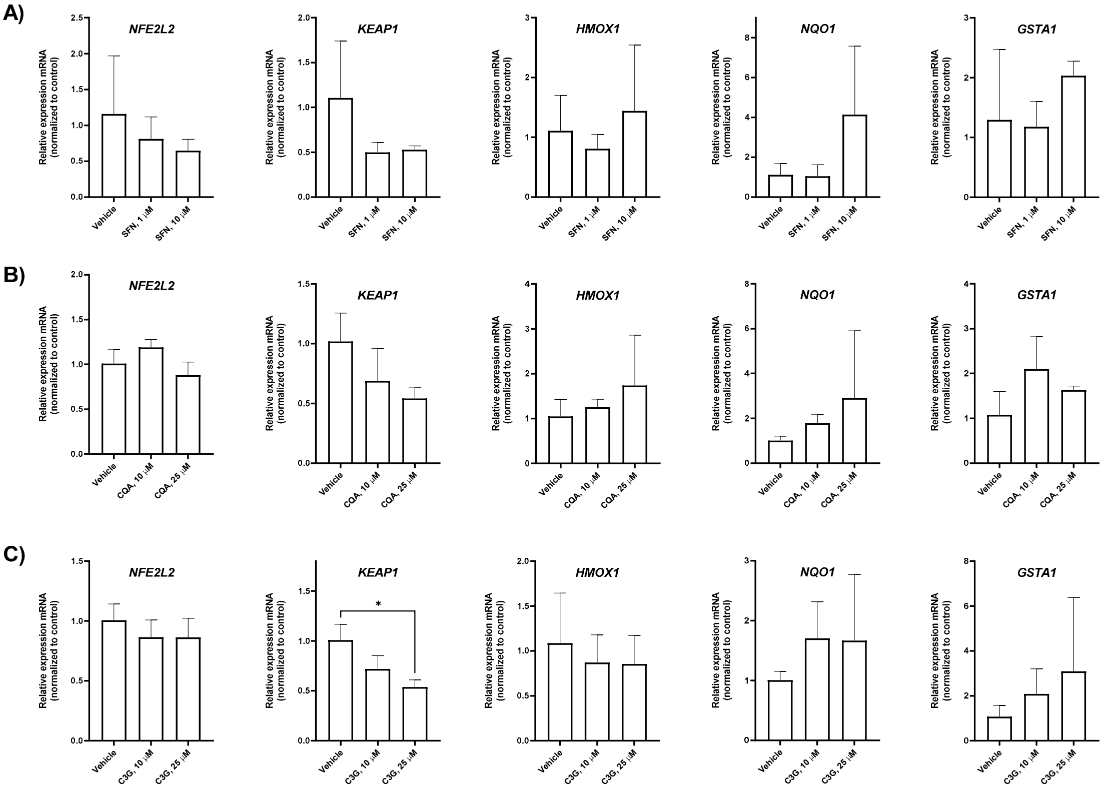

Figure S3. Changes in expression of NRF-2 pathway genes in differentiated HL-60 cell line exposed to different concentrations of A) Sulforaphane (SFN); B) Chlorogenic acid (CQA); and C) Cyaniding-3glc (C3G). All values are represented as the fold change of relative RNA expression between the condition and the vehicle group. Statistical analyses were performed using Kruskal- Kruskal–Wallis one-way analysis of variance and Dunn's *post hoc* analysis. All values are given as mean ± SD. $*p < 0.05$.

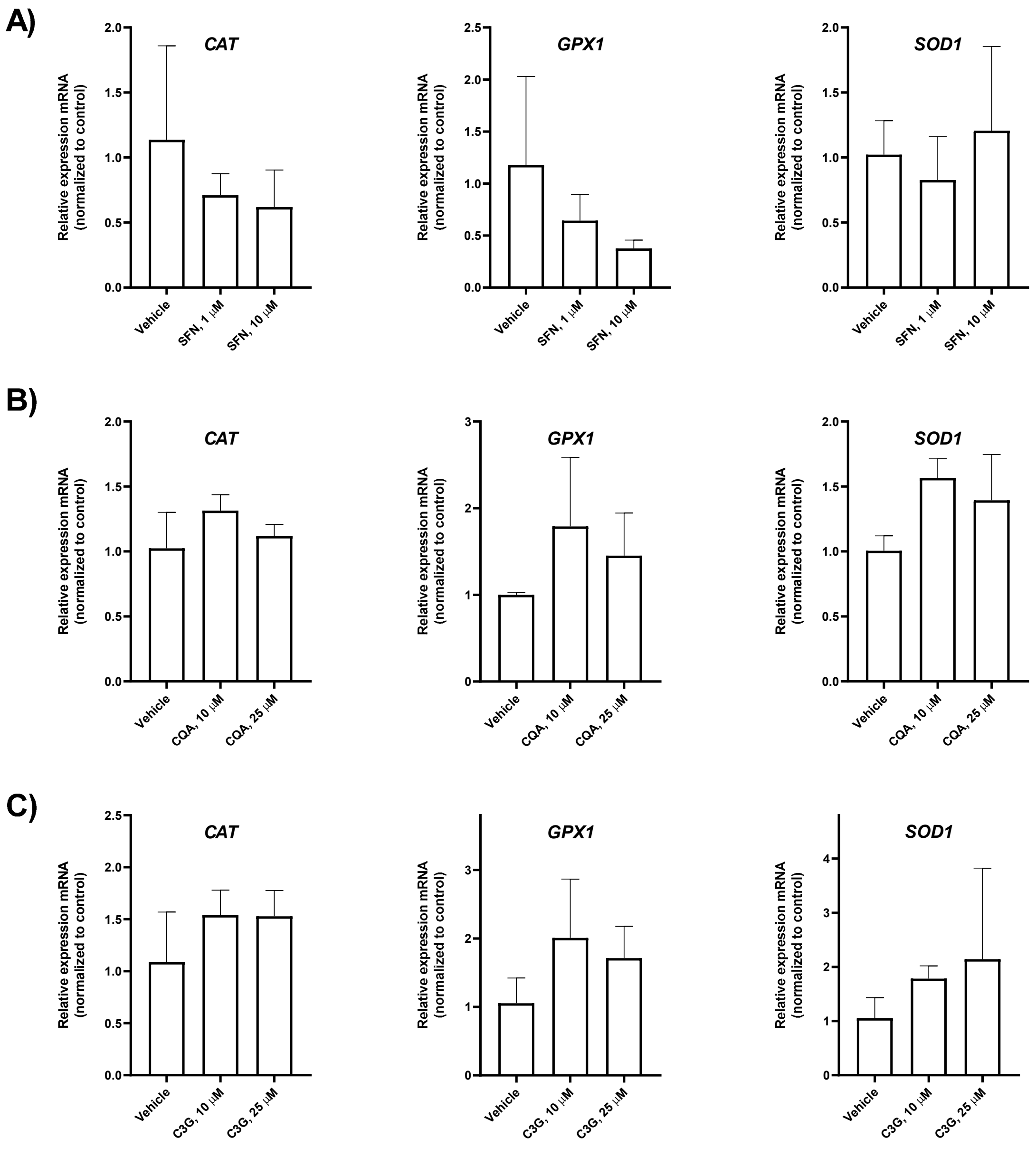

Figure S4. Changes in expression of antioxidant enzyme genes in differentiated HL-60 cell line exposed to different concentrations of A) Sulforaphane (SFN); B) Chlorogenic acid (CQA); and C) Cyaniding-3glc (C3G). All values are represented as the fold change of relative RNA expression between the condition and the vehicle group. Statistical analyses were performed using Kruskal- Kruskal–Wallis one-way analysis of
